# A comparison of the temporary placement of 3 different self-expanding stents for the treatment of refractory benign esophageal strictures: a prospective multicentre study

**DOI:** 10.1186/1471-230X-12-70

**Published:** 2012-06-12

**Authors:** Jorge Manuel Tavares Canena, Manuel José Antunes Liberato, Ricardo António Natário Rio-Tinto, Pedro Miguel Pinto-Marques, Carlos Manuel Menezes Romão, António Vasco Mello Pereira Coutinho, Beatriz Alda Henriques Costa Neves, Maria Filipa Costa Neves Santos-Silva

**Affiliations:** 1 Department of Gastroenterology, Pulido Valente Hospital, Faculty of Medical Sciences, Alameda das Linhas de Torres n° 117, 1769-001, Lisbon, Portugal; 2 Center of Gastroenterology, Cuf Infanto Santo Hospital, Travessa do Castro n° 3, 1350-070, Lisbon, Portugal; 3 Department of Gastroenterology, Santo António dos Capuchos Hospital, Alameda Santo António dos Capuchos, 1169-050, Lisbon, Portugal; 4 Department of Gastroenterology, Garcia de Orta Hospital, Avenida Torrado da Silva, Pragal, 2801-951, Almada, Portugal; 5 Catolica Lisbon School of Business & Economics, Palma de Cima, 1649-023, Lisbon, Portugal

**Keywords:** Refractory benign esophageal strictures, Fully covered self-expanding metal stents, Biodegradable stents, Self-expanding plastic stents, Expandable esophageal stents

## Abstract

**Background:**

Refractory benign esophageal strictures (RBESs) have been treated with the temporary placement of different self-expanding stents with conflicting results. We compared the clinical effectiveness of 3 types of stents: self-expanding plastic stents (SEPSs), biodegradable stents, and fully covered self-expanding metal stents (FCSEMSs), for the treatment of RBES.

**Methods:**

This study prospectively evaluated 3 groups of 30 consecutive patients with RBESs who underwent temporary placement of either SEPSs (12 weeks, n = 10), biodegradable stents (n = 10) or FCSEMSs (12 weeks, n = 10). Data were collected to analyze the technical success and clinical outcome of the stents as evaluated by recurrent dysphagia, complications and reinterventions.

**Results:**

Stent implantation was technically successful in all patients. Migration occurred in 11 patients: 6 (60%) in the SEPS group, 2 (20%) in the biodegradable group and 3 (30%) in the FCSEMS group (*P* = 0.16). A total of 8/30 patients (26.6%) were dysphagia-free after the end of follow-up: 1 (10%) in the SEPS group, 3 (30%) in the biodegradable group and 4 (40%) in the FCSEMS group (*P* = 0.27). More reinterventions were required in the SEPS group (n = 24) than in the biodegradable group (n = 13) or the FCSEMS group (n = 13) (*P* = 0.24). Multivariate analysis showed that stricture length was significantly associated with higher recurrence rates after temporary stent placement (HR = 1.37; 95% CI = 1.08-1.75; *P* = 0.011).

**Conclusions:**

Temporary placement of a biodegradable stent or of a FCSEMS in patients with RBES may lead to long-term relief of dysphagia in 30 and 40% of patients, respectively. The use of SEPSs seems least preferable, as they are associated with frequent stent migration, more reinterventions and few cases of long-term improvement. Additionally, longer strictures were associated with a higher risk of recurrence.

## Background

Benign esophageal strictures are often caused by esophageal reflux, the ingestion of caustic substances, esophageal surgery, and radiation therapy [1-7]. These patients most commonly present with dysphagia [3,5]. Treatment of benign esophageal strictures with serial endoscopic dilatation using bougies or balloons has been established as a standard therapy [2,3,6], and it is associated with an immediate 80-90% success rate of relieving dysphagia [3,6]. However, 30-60% of benign strictures will recur during long-term follow-up. Furthermore, in complex strictures that are usually longer (>2 cm), tortuous, angulated or have a severely narrowed diameter, the recurrence rate is considerably higher [3,5,7]. In these patients, repeated sessions of dilatation without long-term clinical success are associated with increased the risk of developing complications, patient discomfort and recurrent dysphagia; thus, an alternative treatment strategy should be considered [2,3,7]. Surgical procedures, including gastric pull-up and enteral replacement, are potentially curative, but they are associated with high rates of morbidity and mortality, and many patients are not be good surgical candidates or willing to undergo surgery [1,3,6]. Theoretically, temporary placement of expandable esophageal stents permits a longer-lasting dilatation effect, maintains luminal patency and simultaneously stretches the stricture [3-6]. Previous studies have proposed the use of temporary self-expandable metal stents (SEMS) with discouraging results due to the high rate of complications, including embedding of the uncovered metal portion of the stent in the esophageal wall, new stricture formation, necrosis and ulcerations from the stents themselves [2-9].

To avoid complications of partially covered/uncovered stents, temporary placement of 3 different types of expandable stents have been used for the treatment of refractory benign esophageal strictures (RBES): self-expanding plastic stents (SEPSs), biodegradable stents and fully covered SEMSs. Several studies have evaluated the clinical effectiveness of SEPSs [2,4,6,10-17]. Although initial studies with SEPSs showed promising results pertaining to dysphagia relief in the great majority of patients [10,11], recent studies have shown less favorable outcomes, with a clinical success rate well below 50% [2,4,6,12-14]. Furthermore, SEPS are also associated with various complications, especially high stent migration rates [2,11,13,14]. Biodegradable stents have recently been developed and can serve as an alternative for SEPS. Saito et al. reported results from 2 series of patients who received poly-l-lactic esophageal stents [18,19]. In one study Saito et al. observed a high migration rate (10/13 cases), although no symptoms of re-stenosis were observed within the follow-up period in all cases [19]. Three recent studies have used a novel stent (Ella esophageal stent) composed of the biodegradable polymer polydiaxone [4,20,21]. In these 3 studies authors observed low migration rates with the Ella stent (range: 0–22.2%), and encouraging clinical results with clinical success rates ranging from 33% to 60%. One study comparing the Ella stent with the Polyflex stent observed similar clinical performance and complication rates between the 2 stents as well as a significantly lower reintervention rate for the Ella stent group [4]. Finally, an alternative to SEPSs and biodegradable stents in the treatment of RBES is offered by fully covered SEMSs [1,3,22,23]. However, a recent meta-analysis [24] did not include studies with biodegradable stents, and only one study compared SEPSs and biodegradable stents [4]. Thus, no study has included or compared the 3 types of stents available for the treatment of RBES, and no case series have addressed the outcomes of patients treated with a fully covered Wallflex stent.

This study compared 3 groups of consecutive patients with RBES who underwent temporary placement of SEPSs, biodegradable stents and fully covered SEMSs. Data were collected to analyze the technical success and clinical outcomes of the stents as evaluated by recurrent dysphagia, complications and reinterventions.

## Methods

### Patients and setting

Between July 2005 and March 2011, 3 consecutive cohorts of patients with RBES were enrolled in the study and followed prospectively. One of 3 different types of stents were placed, at different times, in 30 consecutive patients: a SEPS, a biodegradable stent or a fully covered SEMS. The stent used was chosen accordingly with the practice at that time in the participating centre. The placement of the stents was done consecutively (e.g. 10 SEPS, then 10 biodegradable stents and then 10 fully cover SEMS), and not at the discretion of the endoscopist. Patients with esophageal fistulas or leaks, suspicion of malignancy or an upper esophageal sphincter within 3 cm of the stricture were excluded from the study. This study was conducted at a total of 4 referral academic centres (Pulido Valente Hospital, Faculty of Medical Sciences, Lisbon, Portugal; Cuf Infanto Santo Hospital, Lisbon, Portugal; Santo António dos Capuchos Hospital, Lisbon, Portugal; and Garcia de Orta Hospital, Almada, Portugal). All patients provided informed written consent prior to undergoing stent placement. Each institutional review board involved approved this study.

### End points and definitions

The primary end point was clinical success defined as the resolution of dysphagia symptoms after the end of long-term follow-up (grade 0–1). Secondary end points included technical success, safe removal of the stents, complications and the need for reinterventions. Dysphagia was graded using a previously described scale as follows: grade 0 = able to eat normal diet/no dysphagia; grade 1 = able to swallow some solid foods; grade 2 = able to swallow only semi solid foods; grade 3 = able to swallow liquids only; grade 4 = total dysphagia [25]. RBES were defined, using a previously defined definition, as the inability to achieve a lumen diameter of 14 mm over five sessions at 2-week intervals or as a result of an inability to maintain a satisfactory luminal diameter for 4 weeks once the target diameter of 14 mm had been achieved [26]. This definition applies only when there is no inflammation at the stricture site. Technical success or successful stent placement was defined as deployment of the stent across the lesion with patency visualized both fluoroscopically and endoscopically. Complications were defined as any adverse event related to stent placement. Complications were also categorized as major (severe events such as perforations, hemorrhage necessitating transfusion, severe pain, or aspiration) and minor (non-life-threatening, such as mild chest pain, globus, reflux symptoms, stent migration, or tissue hyperplasia). Stent migration was defined as either radiographic evidence of the stent within the stomach or endoscopic visualization of the stent having moved from the initial placement location. Reintervention was defined as any procedure performed after initial stent placement, which included stent removal or stent repositioning due to migration, dilatation, additional stent placement and surgery, but did not include scheduled stent removal.

### Technique and stents

Before stent placement, all patients underwent an esophagogram. All procedures were performed with patients in the left lateral position under sedation with propofol administered by an anesthesiologist. A guidewire was passed through the stricture, and dilatation was performed as needed using either a balloon dilator or a Savary-Guilliard dilator at the discretion of the operator. The RBES were dilated large enough only to allow passage of the stent delivery apparatus (e.g. 12–14 mm for the SEPS, 9.4 mm for the Ella stent and 6.2 mm for the fully covered SEMS). After dilatation, if needed, internal radiopaque markers were placed at the distal and proximal edges of the stricture. Stents were deployed under fluoroscopic guidance. Endoscopy was repeated immediately after stent placement for the purpose of visualizing the proximal part of the stent to assess adequate placement and deployment of the stent. Resumption of oral intake was permitted on the same day, and patients were cautioned about aspiration risk when necessary. All patients were placed on proton pump inhibitors after stent placement.

**Figure 1:**
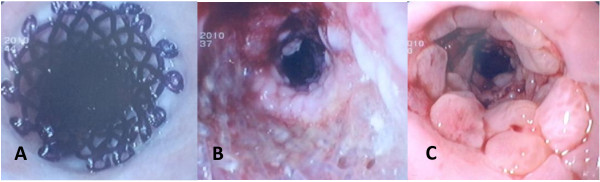
(a) Endoscopic view of a biodegradable stent immediately after placement. (b) Endoscopic appearance of the process of biodegradation at 2 months. (c) Endoscopic appearance of tissue hyperplasia 3 months after stent degradation.

**Figure 2:**
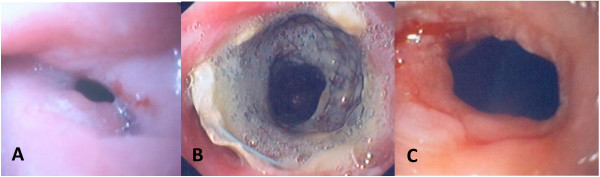
(a) Endoscopic view of an anastomotic stricture before stent placement. (b) Endoscopic view of a fully cover SEMS 3 months after stent deployment. (c) Endoscopic appearance of the initial stricture 3 months after stent removal.

The SEPS used in this study was the Polyflex stent (Boston Scientific, Natick, Massachusetts). This stent is made of polyester netting and is completely covered with a silicone membrane. The proximal end of the stent is flared (4 and 5 mm larger than the body diameter) to prevent stent migration. The stent is manually mounted on a delivery system ranging from 12–14 mm (36–42 French) depending on the stent diameter. The Polyflex stent is available in 3 lengths (10, 12 and 15 cm) and 3 body diameters (16, 18 and 21 mm). Radiopaque markers are placed at both ends and in the middle to aid fluoroscopic visualization. The biodegradable stent used was the Ella stent (Ella-CS, Hradec Kralove, Czech Republic), which is manufactured from commercially available polydioxanone absorbable surgical suture. The stent is not removable, is radiolucent and has radiopaque markers at both ends and in the middle to aid fluoroscopic visualization. The stent must be manually loaded onto a 9.4 mm (28 French) delivery system immediately before implantation. The Ella stent is available in 4 body diameters (18, 20, 23 and 25 mm) with lengths ranging from 60 to 135 mm. The stent has flared ends (5 and 6 mm larger than the body diameter) to reduce migration rates. The stent begins to degrade after 4 to 5 weeks, and the degradation process is completed within 2 to 3 months after placement (Figure 1). The fully covered SEMS used was the Wallflex stent (Boston Scientific, Natick, Massachusetts), which is composed of platinol and is entirely covered with a silicone covering. The stent has flared ends (the flare is 5 mm larger than the body diameter) to better assist in anchoring the stent, and the proximal end has a blue removal suture to facilitate stent removal (Figure 2). The stent has a 6.17 mm (18.5 French) delivery system and is available in 3 lengths (10, 12 and 15 cm) and 2 body diameters (18 and 23 mm). We used the largest body diameter available in all cases: 21 mm for SEPS, 25 mm for biodegradable stents and 23 mm for fully covered SEMS. The removal of plastic and metal stents was planned at 3 months after placement in all patients. All stents were removed with rat-tooth forceps.

### Follow-up

The follow-up continued from stent insertion until at least 8 months after stent removal/degradation/migration in all patients. Endoscopies (to detect asymptomatic migration and/or hyperplastic tissue overgrowth), assessments of dysphagia and investigations of any potentially stent-related symptoms were conducted at 1, 2 and 3 months. After stent removal/stent degradation, patients were contacted on a monthly basis and asked to grade their dysphagia using the scale previously described in this manuscript. After 6 months, patients were submitted to endoscopy and then followed on a 2-month basis. In the case of recurrent dysphagia during follow-up, patients contacted their medical assistant in the institution for analysis and reintervention when needed.

### Statistical analysis

The intention-to-treat method was used in all analyses. The *χ*2 test, Kruskal-Wallis test and the Mann–Whitney *U* test were used to calculate the statistical significance of different demographic and clinical variables when appropriate. Dysphagia scores, taken at baseline, at 4 weeks and after stent removal/dysphagia recurrence, were compared within each stent group using the Wilcoxon signed-rank test. The dysphagia-free period (esophageal patency) during follow-up after stent removal/degradation/migration was evaluated by the Kaplan-Meier method, and groups were compared using the log-rank test. Multivariate Cox proportional hazard models with forward selection were used to evaluate the multivariate factors potentially affecting the dysphagia-free period after temporary stenting. Age, sex, type of stent, length and location (upper + lower esophagus, middle esophagus or anastomotic) of stricture were the variables included in the analysis. A Poisson regression was conducted to determine possible factors affecting the number of reinterventions. All reported *P*-values were for two-sided test, and a *P*-value less than 0.05 was considered to be statistically significant. Statistical analysis was performed using SPSS (Statistical Package for the Social Sciences) 18 (IBM Corporation, New York, USA).

## Results

### Patients

Between July 2005 and March 2011, 30 patients (16 males and 14 females) with a mean age of 53.5 years (range: 27–79 years) were enrolled in the study. Ten patients were included in each group. Patient demographics, stricture characteristics, indications for stent placement and baseline dysphagia scores are summarized in Table 1. There were no significant differences in the demographics and baseline characteristics among the 3 groups defined earlier in the Methods section.

**Table 1:** Patient demographics, characteristics of strictures, indications for stent placement and baseline dysphagia scores

	**SEPS (n = 10)**	**BD Stent (N = 10)**	**FCSEMS (n = 10)**	** *P* **
Sex, n (%)				0.53
Male	5 (50%)	4 (40%)	7 (70%)	
Female	5 (50%)	6 (60%)	3 (30%)	
Age (years), mean (range)	52.7 (28–67)	57.2 (42–79)	50.7 (27–78)	0.63
Characteristics of stricture				
Lenght (cm), mean (range)	2.9 (1–5)	2.9 (1–8)	2.8 (1–6)	0.91
Location, n (%)				0.14
Upper Esophagus	-	-	2 (20%)	
Mid esophagus	2 (20%)	-	2 (20%)	
Lower esophagus	4 (40%)	4 (40%)	3 (30%)	
Anastomotic	4 (40%)	6 (40%)	3 (30%)	
Indication for stent placement,				0.2
n (%)				
Peptic stricture	1 (10%)	3 (30%)	3 (30%)	
Caustic	1 (10%)	1 (10%)	1 (10%)	
Radiation induced	2 (10%)	-	-	
Post-surgical	4 (40%)	6 (10%)	3 (30%)	
Idiophatic	2 (20%)	-	3 (30%)	
Dysphagia score before stent placement, mean (±SD)	2.8 (0.42)	2.8 (0.42)	2.7 (0.48)	0.84

SEPS Self-expandable plastic stent, BD Biodegradable, FCSEMS Fully cover self-expandable metal stent.

### Stent placement, removal and degradation

Stent implantation was technically successful (Table 2) in all patients without procedure-related complications. Migration occurred, as discussed later, in 11 patients: 6 in the SEPS group, 2 in the biodegradable stent group and 3 in the fully covered SEMS group. At the 3-month scheduled endoscopy, biodegradable stents that were still in place appeared to be almost dissolved. At the 6-month endoscopy, there were no traces of the previously placed biodegradable stents. All SEPSs and fully covered SEMSs that had migrated into the stomach were subsequently removed or repositioned. Migrated biodegradable stents were left to fragment in the stomach and were not associated with any symptoms or complications. SEPS and fully covered SEMS that remained in position for the intended 3-month temporary placement were retrieved successfully with no procedural complications.

**Table 2:** Technical success, clinical outcome, dysphagia evolution and reinterventions after temporary placement of 3 different self-expanding stents for the treatment of refractory benign esophageal strictures

	**SEPS (n = 10)**	**BD Stent (N = 10)**	**FCSEMS (n = 10)**	** *P* **
Technical success, n (%)	10 (100%)	10 (100%)	10 (100%)	-
Clinical success	1 (10%)	3 (30%)	4 (40%)	0.27
Dysphagia score, mean (±SD)				
At 4 weeks	0.7 (0.48)	0.4 (0.52)	0.5 (0.53)	0.4
Post-stenting	2.4 (1.26)	2.0 (0.82)	1.6 (1.26)	0.23
Time to dysphagia recurrence (weeks), mean (range)	4.3 (2–9)	3.8 (2–9)	3.8 (2–8)	0.75
Reinterventions, n	24	13	13	0.24
Final outcome, n (%)				-
Successful treatment	1 (10%)	3 (30%)	4 (40%)	
Repeat stenting	2 (20%)	1 (10%)	3 (30%)	
Dilatations	1 (10%)	3 (30%)	2 (20%)	
Surgery	6 (60%)	3 (30%)	1 (10%)	
Follow-up (months), median (range)	43 (16–66)	18.5 (11–21)	10 (8–12)	-

SEPS Self-expandable plastic stent, BD Biodegradable, FCSEMS Fully cover self-expandable metal stent, Time to dysphagia recurrence in patients without clinical success, Post-stenting score in all patients after dysphagia recurrence/long-term free of dysphagia.

### Clinical effectiveness and evaluation of dysphagia

**Figure 3:**
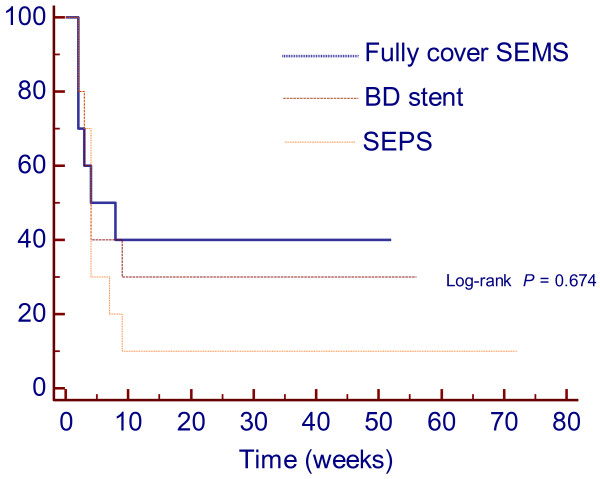
**Cumulative period free of dysphagia (esophageal patency) curves by Kaplan-Meier analysis in patients treated using self-expandable plastic stent (SEPS), biodegradable (BD) stent and fully cover self-expandable metal stent (SEMS).** Although not statistically significant there is an advantage using fully cover SEMS and BD stent vs. SEPS. The benefits of stenting decreased rapidly with time and esophageal patency appeared to plateau between 8–9 weeks for all stents.

**Figure 4:**
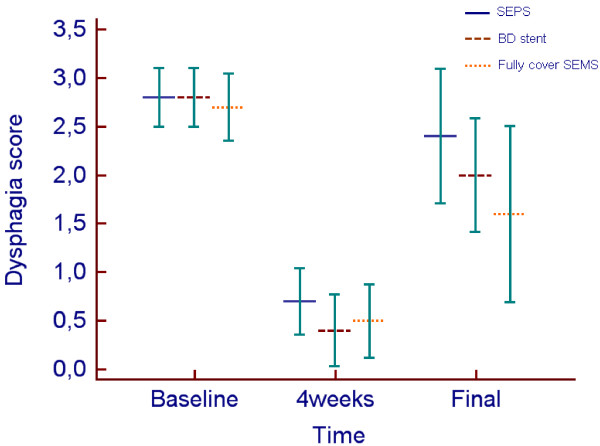
**Clustered multiple variables graph with mean dysphagia scores over time after temporary placement of self-expandable plastic stent (SEPS), biodegradable (BD) stent and fully cover self-expandable metal stent (SEMS).** Bars represent 95% CI for mean. Dysphagia score improved significantly from baseline level to 4 weeks after stent placement (p < 0.001) in the 3 groups. Dysphagia score improved significantly from baseline level to post treatment for BD stent and fully cover SEMS but not for SEPS. (final post-treatment after stent removal and dysphagia recurrence for all patients).

Stent placement outcomes are shown in Table 2. Overall, regarding clinical success, a total of 8/30 patients (26.6%) who received temporary self-expandable stents were dysphagia-free after a median follow-up time of 23.4 months (range: 8–66 months). In the SEPS group, 1 patient (10%) was dysphagia-free after a median group follow-up time of 42.7 months (range: 16–66 months). Following temporary placement of a biodegradable stent, 3 patients (30%) were dysphagia-free after a median follow-up time of 18.5 months (range: 11–21 months). Of the 10 patients treated with fully covered SEMS, 4 (40%) were dysphagia-free after a median follow-up time of 10 months (range: 8–12 months). There were no significant differences in the clinical successes of the 3 types of stents (P = 0.27) [SEPS vs. biodegradable stent (*P* = 0.58); SEPS vs. fully covered SEMS (*P* = 0.30); biodegradable stent vs. fully covered SEMS (*P* = 0.64)]. Kaplan-Meier analysis (Figure 3) showed that the cumulative period of being dysphagia-free (esophageal patency) during follow-up was not significantly longer in patients treated with SEPS (mean 11.1 months, 95% CI 0.0-23.75), biodegradable stents (mean 19.5 months, 95% CI 4.64-34.36) or fully covered SEMS (mean 23.1 months, 95% CI 8.44-37.76) (*P* = 0.67). The estimated relative risk of dysphagia recurrence [hazard ratio (HR)] was 1.34-fold higher in the SEPS group than in the biodegradable stent group (95% CI 0.50-3.58). Temporary placement of SEPSs resulted in a HR in these patients that was 1.6-fold higher than in patients who submitted to temporary fully covered SEMS placement (95% CI 0.58-4.41). The HR was 1.15-fold higher in the biodegradable group than in the fully covered SEMS group (95% CI 0.39-3.41). Multivariate Cox hazard analysis showed that the length of stricture (HR = 1.37; 95% CI 1.08-1.75; *P* = 0.011) was the only factor associated with the period of dysphagia-free after temporary stent placement. Longer strictures had at higher risk of recurrence. Dysphagia score evolution for the 3 groups is shown in Figure 4. Dysphagia scores improved significantly from the pre-treatment scores to the scores at 4 weeks after stent placement (*P* < 0.001) in the 3 groups. However, when comparing the dysphagia scores at baseline and after stent removal/dysphagia recurrence, differences were significant in the biodegradable stent group (2.8 ± 0.42 vs. 2.0 ± 0.82; *P* = 0.02) and fully covered stent group (2.7 ± 0.48 vs. 1.6 ± 1.26; *P* = 0.008), but these differences were not observed in the SEPS group (2.8 ± 0.42 vs. 2.4 ± 1.26; *P* = 0.10).

### Complications

Complications (Table 3) occurred in 7 patients (9 complications) who received a Polyflex stent, in 5 patients (7 complications) who received an Ella stent and in 6 patients (6 complications) who received a Wallflex stent (P = 0.38). In the SEMS group, 1 patient experienced both moderate chest pain and reflux symptoms, and another patient had moderate chest pain before stent migration. In the biodegradable stent group, 1 patient experienced severe chest pain before stent migration, and 1 patient with tissue ingrowth experienced major bleeding. Minor complications (n = 9) occurred more frequently after SEPS placement than after biodegradable stent placement (n = 5) or fully covered SEMS placement (n = 6), although this difference was not statistically significant (*P* = 0.21). Globus sensation occurred in 1 patient after treatment with a Wallflex stent. Although the proximal flare of the stent was positioned 4 cm below the upper esophageal sphincter, the patient experienced an increasing globus sensation leading to early removal of the stent (3 weeks). Importantly, tissue hyperplasia (ingrowth) was observed in 3 patients submitted to temporary biodegradable stent placement, and was not observed in the patients submitted to treatment with SEPS or with fully covered SEMS. The statistical value for the comparison between tissue hyperplasia with the Ella stent compared to the other 2 stents was *P* = 0.086. Hyperplasia was associated with major bleeding in 1 patient and with recurrent dysphagia in 2 patients. Migration occurred more frequently after SEPS placement than after biodegradable stent placement or fully covered SEMS placement, although this difference was not statistically significant (*P* = 0.16); [SEPS vs. biodegradable stent (*P* = 0.06); SEPS vs. fully covered SEMS (*P* = 0.17); biodegradable stent vs. fully covered SEMS (P = 0.61)]. Stent migration was observed in 6 patients at 2, 3, 4, 5, 6 and 6 weeks after SEPS placement. Migration occurred in 2 patients at 3 and 4 weeks after biodegradable stent placement and in 3 patients at 2, 3 and 4 weeks after temporary placement of a fully covered SEMS. Major complications occurred in 2 patients after biodegradable stent placement. One patient developed severe thoracic pain requiring treatment with morphine, and the other patient developed heavy bleeding with a significant drop in hemoglobin level. The latter patient was admitted to receive a blood transfusion, and the hemorrhage stopped without further intervention.

**Table 3:** Minor and major complications after temporary placement of 3 different self-expanding stents for the treatment of refractory benign esophageal strictures

**Complications**	**SEPS (n = 10)**	**BD Stent (N = 10)**	**FCSEMS (n = 10)**	** *P* **
Total, n in number of patients, n (%)	9 in 7 (70)	7 in 5 (50)	6 in 6 (60)	0.38
Minor, n (%)	9 (90)	5 (50)	6 (60)	0.21
Globus sensation	0	0	1	-
Stent migration	6	2	3	0.16
Reflux symptoms	1	0	1	-
Moderate chest pain	2	0	1	-
Tissue hyperplasia (in or overgrowth)	0	3	0	0.086
Major, n (%)	0	2 (20)	0	-
Hemorrhage	0	1	0	-
Severe chest pain	0	1	0	-

SEPS Self-expandable plastic stent, BD Biodegradable, FCSEMS Fully cover self-expandable metal stent.

### Follow-up and reinterventions

Overall, 22 patients (73.3%) were submitted to reinterventions (Table 2), which included dilatation, repeat stenting and surgery. More reinterventions were required in the SEPS group (n = 24) than in the biodegradable stent group (n = 13) or the group with temporary placement of a fully covered SEMS (n = 13). These differences were not significant based on the Kruskal-Wallis test (*P* = 0.24). However, Poisson regression showed that fully covered SEMSs and biodegradable stents resulted in a significantly lower number of reinterventions (B = −0.87; *P* < 0.05). Migration was observed in 6 patients after SEPS placement. One patient underwent surgery, and the other 5 patients underwent further stenting with either SEPSs (3 patients, 7 stents) or fully covered SEMSs (2 patient, 2 stents). In the 3 patients who were submitted to further stenting with SEPSs, the number 7 stents refers to the total number of stent re-placements; in these patients we did not place a new stent, but we repositioned stents that had migrated. Two patients are still being treated with temporary stent placement (fully covered SEMSs, which migrated again and were repositioned), and 3 patients underwent surgery. Three patients had their SEPSs removed after 3 months and developed recurrent dysphagia. One is still being treated with repeat dilatations, and 2 required surgery. After surgery, one patient had a post-surgical stricture successfully managed by repeated dilatations. In the biodegradable group, stent migration was observed in 2 patients. One patient required surgery, and a post-surgical stricture developed after 3 months. The patient was unsuccessfully managed with a biodegradable stent and is currently under monthly dilatation therapy. The other patient was submitted to repeat dilatations and finally to further stenting with a fully covered SEMS, which migrated into the stomach and was repositioned twice. There were five patients in whom a biodegradable stent completely dissolved in place; they each presented with recurrent dysphagia. Two required surgery and were dysphagia-free (score 0–1), and the remaining 3 are still being treated with repeat dilatations. Stent migration occurred in 3 patients after temporary placement of a fully covered SEMS. Two patients were submitted to further stenting with a fully covered SEMSs (2 stents per patient with new migrations and repositioning of the stent), and the third patient is being treated with repeat dilatations. Of the 3 patients with successful fully covered SEMS removal (one patient had his stent removed at 3 weeks due to globus) and dysphagia recurrence, one patient was further treated with another fully covered stent for 6 months and was dysphagia-free (score 0–1) at 12 weeks after removal of the second stent. One patient was treated with repeat dilatations, and the remaining patient required surgery and is dysphagia-free (score 0–1). For the 22 patients in whom clinical success was not achieved, the mean time to dysphagia recurrence after stent removal/migration/degradation was 4 weeks (range: 2–9 weeks), and there were no significant differences in the time to recurrence in patients treated with SEPSs (mean: 4.3 weeks, range: 2–9 weeks), biodegradable stents (mean: 3.8 weeks, range: 2–9 weeks) or fully covered SEMSs (mean: 3.8 weeks, range: 2–8 weeks) (*P* = 0.75) [SEMS vs. biodegradable stent (*P* = 0.95); SEPS vs. fully covered SEMS (*P* = 0.61); biodegradable stent vs. fully covered SEMS (*P* = 0.54)].

## Discussion

According to our findings in a prospective, nonrandomized study of 3 consecutive cohorts of patients with RBES, temporary placement of a biodegradable stent or a fully covered SEMS may lead to a long-term dysphagia-free period in 30 and 40% of patients, respectively. The use of SEPS seems to be least preferable, as it is associated with frequent stent migration, more reinterventions and few cases (10%) of long-term improvement. A long stricture was the only significant factor associated with a higher recurrence rate after temporary stent placement.

Unfortunately, unsuccessful management of benign esophageal strictures by serial endoscopic dilatation using bougies or balloons occurs, and the management of these refractory strictures has been considered challenging [4,5,7,27]. Surgery can provide definitive treatment but has been associated with considerable mortality and morbidity, including the development of new anastomotic strictures [2-9]. Partially covered metal stents, which are highly effective in the palliation of malignant esophageal strictures, are associated with high complication rates and are not recommended for RBES [28]. Thus, in a group of patients who have few treatment options available, temporary stent placement can be considered, especially if the procedure is associated with a low complication rate. However, stent placement should be reserved for true refractory strictures, as defined earlier in the Methods section [26]. In our study, all patients were first submitted to serial dilatations during a number of sessions ranging from at least 5 to 10 attempts (some patients were referred from community hospitals where the number of dilatations was greater than 20). Furthermore, all patients were further submitted to several sessions (3 to 6) of intralesional 4-quadrant steroid injections. As proposed by Siersema et al. [4,5,7,27], temporary stent placement was a late step in the treatment strategy of benign esophageal strictures.

There are several case series that have evaluated the outcomes of SEPS with conflicting results (Table 4). Some initial studies have shown good results with large numbers of patients (76.5-94%) being dysphagia-free at the end of follow-up and few complications [10,11,15]. However, more recent studies have shown less favorable outcomes, tempering the initial enthusiasm, with success rates between 0 and 30% [2,6,12-14].

**Table 4:** Case series evaluating the outcomes of self-expandable plastic stents for benign esophageal diseases

**Author (reference**)	**Patients (n)**	**Esophageal strictures (n)**	**Clinical success n (%)**	**Complications**
Evrard et al. [11]	21	17	13/17 (76.5%)	Migration 9/17 (52.9%)
Repici et al. [10]	15	15	12/15 (80%)	Migration 1/15 (6.7%)
Martin et al. [15]	42	18	17/18 (94.4%)	Migration 1/18 (5.5%)
Holm et al. [2]	30	22	5/83 interventions (6%)	Migration 53/83 stents placed (63.9%)
Dua et al. [6]	40	40	12/40 (25%)	Migration 8/40 (22%) Perforation 2/40 (5%) Bleeding 3/40 (7.5%) Mortality 1/40 (2.5%)
Oh et al. [12]	13	13	3/13 (23%)	Migration 4/13 (30.8%)
Triester et al. [13]	5	5	0/5 (0%)	Migration 3/5 (60%) Perforation 1/5 (20%) Severe chest pain 1/5 (20%)
Barthel et al. [14]	8	8	1/8 (12.5%)	Migration 11/13 (85%)

Regarding clinical success of SEPS, the results of our study are clearly in line with those of recent studies [2,6,12-14]. At the end of the follow-up period, only 1 patient was dysphagia free. Migration was observed in 60% of our patients. Again, this is in accordance with some studies that have reported migration rates between 52.9 and 85% [2,11,13,14]. One explanation for our high migration rate could be the duration of the study (3 months); longer stenting times are associated with higher risks of migration [2,11]. In our study, half (n = 3) of the patients had a stent migration after 4 weeks. Alternatively, another explanation could be associated with the result that 8 patients had strictures located in the distal esophagus or in anastomotic areas, as these locations have been associated with higher migration rates for SEPS [2]. Clinically significant tissue hyperplasia was not observed in our study as one might expect from a fully covered device, and this result is in line with the literature [2,4,6,10-16] and also with a recent pooled analysis where tissue in- and overgrowth was uncommon [17].

Theoretically, a biodegradable stent is ideal for the treatment of RBES as it becomes embedded in the esophageal wall, reducing migration rates, dissolving spontaneously after placement and decreasing the need for reinterventions to remove the stent. Furthermore, a stent made from a material with good tissue compatibility should overcome/reduce the in- and overgrowth of reactive tissue. Three recent studies have reported preliminary results with the new Ella stent [4,20,21]. An initial prospective study with 21 patients demonstrated a long-term improvement in 9/21 of the patients (42.9%) [20]. Migration occurred in 2 patients (9.5%), and clinically significant tissue hyperplasia was observed in 1 patient (4.75%). Severe post-stenting pain developed in 3 patients, and minor bleeding was observed in 1 patient. In a prospective study comparing Polyflex stents with ELLA stents, the temporary placement of a biodegradable stent was curative in 6/18 patients (33%) [4]. Major complications occurred in 4 patients (2 had severe retrosternal pain and 2 had hemorrhaging). Clinically significant tissue in- and overgrowth was observed in 2/18 patients (11%). A recent prospective case series of 10 patients who underwent Ella stent placement for benign esophagogastric anastomotic strictures showed a clinical success rate of 60% [21]. No migration was observed, and no complications were observed. Signs of tissue hyperplasia were noticed in 4 patients; however, this was associated with reobstruction and symptoms of dysphagia in only 2 patients.

In our study, 30% of patients in the biodegradable stent group were dysphagia-free at the end of the follow-up period, which is in accordance with 2 previously described studies that have reported a clinical success between 33 and 42.9% [4,20]. Our migration rate was 20%; however, although this was similar to the rate reported by van Boeckel et al. [4], this result was unexpected. We anticipated that the embedding of the uncovered stent into the esophagus would prevent migration. In our study, migration occurred in 2 initial patients, and both had long strictures. Eventually, it was found that length selection of the stent was not the best preventative measure of early migration. After the 2 episodes of migration, we began to perform larger balloon dilatation of the stent immediately after deployment to further embed the stent into the esophageal mucosa and reduce the risk of migration. The implementation of balloon dilatation of the Ella stent after deployment did decrease migration rates compared to before balloon dilatation. No other cases of migration were observed and we suggest that this measure can help to mitigate migration after Ella stent deployment. Clinically significant hyperplasia (ingrowth) was observed in 3/10 patients (1 bleeding, 2 reobstructions); this rate was slightly higher than the rates from the 3 studies previously described [4,20,21]. The uncovering of the stent may facilitate hyperplasia along with the eventual chemical reaction of the esophageal mucosa with the polydioxanone. However, the low rate of in- and overgrowth found in the literature and in this study suggests that the biodegradable stent has good tissue compatibility.

Removable, fully covered SEMS are an alternative to SEPS and biodegradable stents. Their fully covered design is thought to induce less reactive hyperplasia, making these stents easier to remove and facilitating stenting over longer periods [3,23,28]. Few published studies, mostly retrospective, have looked at the outcomes of the use of fully covered SEMSs, and they have reported variable data. One interventional radiology study evaluated the effectiveness of temporary stenting in 55 patients [1]. Three different types of stents were left in place from 1 week to 6 months, and long-term symptom relief was reported in 31% (17/55) of patients. Migration and tissue hyperproliferation were observed in 25 and 31% of patients, respectively. Using the Alimaxx stent, Eloubeidi et al. reported their results from a study of 19 patients [3]. Clinical success was achieved in 21% of patients, no tissue hyperplasia was reported and migration rates were 37%. A recent study retrospectively reviewed 24 patients with refractory post-esophagogastric anastomosis strictures [23]. The stents were removed within 4–8 weeks after placement, and 17/24 patients (70.8%) were free from dysphagia at the end of the follow-up period (12 months) as evaluated by intention-to-treat analysis. Tissue hyperplasia was not observed, and migration was observed in only 1 patient (4.2%). Well-tolerated chest pain and reflux symptoms were the most frequent complications reported.

Our study is the first prospective case series to report the outcomes of treatment with a fully covered Wallflex stent. This stent is easy to implant and can be safely removed. However, complications do occur, and migration was the most common complication observed. Migration is frequent for fully covered stents because of the reduced anchoring capacity of these devices [1,3,4,28]. Migration occurred in 3 patients (30%), which is in accordance with the results of 2 previously cited reports (25 and 37%) [1,3]. Compared with the SEPS used in our study, which are also fully covered stents, the migration rate of the Wallflex stent was reduced by a factor of 2 (30% vs. 60%). We believe that this result was related to the larger diameters, stronger radial force and the design of the Wallflex stent, which has 2 flared ends. In contrast, the Polyflex stent has only 1 flared end. Another concern with temporary stent placement is tissue hyperplasia. The fact that these stents do not become embedded in the esophageal wall makes clinically significant tissue in- and overgrowth less likely to occur. In our study of 10 patients, no clinically significant tissue hyperplasia was observed, which is in accordance with other reports [3,23]. Only interventional radiology studies have reported significant tissue hyperplasia [1,22], which should be interpreted with caution because there was no endoscopic visualization of such tissue hyperproliferation. Furthermore, in our study, one patient was submitted to further stenting over 6 months, and, after that period, the stent was safely removed without signs of hyperplasia. Overall, 4 patients (40%) had long-term resolution of their symptoms after stent removal, which is comparable to the results of 4 earlier studies reporting different clinical successes: 31% [1], 21% [3], 48% [22] and 70.8% [23] (average: 42.5%).

Although not statistically significant, the results of biodegradable stents or fully covered SEMSs were superior to those of SEPSs in terms of several variables. For example, the duration that patients were dysphagia-free after temporary stenting with a biodegradable stent or a fully covered SEMS was almost 2-fold higher than after placement of a SEPS. While stents were in place, there was a significant difference in the dysphagia scores compared to baseline scores for all stents; all stents were equally effective. However, when comparing baseline dysphagia to post-treatment scores, a significant difference was found in the fully covered group and the biodegradable group but not in the SEPS group; this is a consequence of the differing clinical success rates of the 3 stents. Overall, in the SEPS group, only 1 patient experienced long-term improvement. An explanation for this could be the high rate of SEPS migration when compared with the other 2 stents used. Patients with fully covered or biodegradable stents had their stents in place for longer periods, which may indicate that longer stenting times correlate with higher success rates [4,10,11]. The optimal time of stenting is unknown and has been a subject of debate as clear guidelines have yet to emerge. In our multivariate analysis, the length of the stricture was the only factor associated with higher recurrence rates after temporary stent placement. Thus, it is possible that longer strictures would require longer treatment as suggested elsewhere [1,7,22,29]. The optimal duration of temporary stenting can also be influenced by the underlying cause of injury or by the time since the injury to the esophagus occurred [22,29].

Stenting is safe. In our study, with the exception of migrations, the number of complications was low and, and the complications that occurred were well tolerated. Only 2/30 patients (6%) had significant complications (1 case of bleeding and 1 case of severe pain), which is consistent with most published studies using different expandable stents [2-4,10-12,14,15,17,20,21,23,24,30]. The safety of the procedure in association with reasonable clinical success in a group of patients that is very difficult to manage makes temporary stent placement an appealing option.

Poisson regression showed that fully covered SEMSs and biodegradable stents resulted in a significantly lower number of reinterventions. More reinterventions were required in the SEPS group, which was due to the higher migration rate along with the lower clinical success rate of SEPSs. One of the reinterventions in all groups was restenting. The clinical effectiveness of restenting is limited. While stents are in place, patients benefit from another dysphagia-free period, but after stent removal, the number of patients who experience long-term clinical success is low. This result is consistent with several studies [1,2,12,14,22,23]. In our study, restenting was performed in 9 patients, and only one was dysphagia-free after the restenting procedure was performed. In those patients whose primary stent migrated, the second stent generally migrated as well, regardless of whether it was the same or a different fully covered stent, which is in line with other studies in the literature [1,2,14,22]. After migration, restenting with fully covered devices is of poor value; therefore, we suggest restenting with a biodegradable stent which seems to be a better option, as it is associated with a lower migration rate due to the potential embedding of the biodegradable stent in the esophagus.

We suggest that further prospective randomized studies are needed to compare biodegradable stents with fully covered SEMS to determine clinical effectiveness, optimal duration of stenting, value of restenting for longer periods, cost and patient satisfaction.

The present study has several limitations. First, our nonrandomized design could have introduced sampling bias. However, there were no major differences in location, etiology or stricture length between the 3 groups studied. Another potential weakness is the different time to follow-up for the 3 groups. The SEPS group had a longer follow-up period, which could have influenced the final outcome because, theoretically, the longer the follow-up period the higher the probability of dysphagia recurrence. However, in our study, all patients had at least 8 months of follow-up after stent removal/degradation/migration, and most other studies showed that the benefits of temporary stenting decreased rapidly with time for non-responders [1,12,20,21,23]. In our study, no patient had recurrent dysphagia after 9 weeks following stent removal/migration/degradation (Figure 3). Thus, different follow-up times were not likely to be associated with the poor results found in the SEPS group. Our study had a relatively low number of patients per group, which limited the study’s statistical strength. However, most of the studies using self-expanding stents have small population sizes due to the low incidence of RBES, and studies with a patient population large enough to have adequate power to detect minor outcome differences are generally impractical. Finally, this study was performed in tertiary referral centers, and we cannot exclude the possibility of obtaining a poorer outcome in community hospitals.

The strengths of our study are the prospective design with a relatively long-term follow-up. To our knowledge, this is the first study to compare the 3 types of self-expanding stents available for the treatment of RBES and the first case series to report the outcomes of patients treated with a fully covered Wallflex stent.

## Conclusions

In conclusion, our data suggest that the temporary placements of biodegradable stents and fully covered SEMSs have similar utility in the treatment of RBES, as they were associated with clinical success in 1/3 of our patients. Of the 3 available stents, SEPSs were associated with the worst clinical success rate as well as with a higher number of migrations and reinterventions. Taken together, temporary stenting for RBES may be useful, especially in those patients in whom other therapeutic options are unavailable. However, better stents and different strategies are needed to overcome the difficulties of managing these patients, particularly when a long stricture is present.

## Competing interests

Jorge Canena is a consultant for Boston Scientific. However, did not receive any financial arrangements related to this research nor any assistance with manuscript preparation. For the remaining authors there are no competing interests.

## Authors’ contributions

Conception and design of the study, performing endoscopies, collection of data, analysis and interpretation of data, and drafting the manuscript: JC; performing endoscopies, collection of data, critical revision of the manuscript and approval of the final draft submitted: ML; performing endoscopies, collection of data, critical revision of the manuscript and approval of the final draft submitted: RRT; performing endoscopies, collection of data, critical revision of the manuscript and approval of the final draft submitted: PPM; performing endoscopies, critical revision of the manuscript and approval of the final draft submitted: CR; performing endoscopies, critical revision of the manuscript and approval of the final draft submitted: AC; critical revision of the manuscript and approval of the final draft submitted: BCN; critical revision of the statistical analysis and approval of the final draft submitted: MFS. All authors read and approved the final manuscript.

## Pre-publication history

The pre-publication history for this paper can be accessed here:

http://www.biomedcentral.com/1471-230X/12/70/prepub
